# A Turn-On Fluorescence Sensor Based on Guest-Induced Luminescence Ru(bpy)_3_^2+^@UiO-66 for the Detection of Organophosphorus Pesticides

**DOI:** 10.3390/molecules30153130

**Published:** 2025-07-25

**Authors:** Jun Li, Jianlan Deng, Qian Tao, Chenyu Yan, Yuxuan Liu, Jianxiao Yang, Zhong Cao

**Affiliations:** 1 Hunan Provincial Key Laboratory of Materials Protection for Electric Power and Transportation, Hunan Provincial Key Laboratory of Cytochemistry, School of Chemistry and Pharmaceutical Engineering, Changsha University of Science and Technology, Changsha 410114, China; 007898@csust.edu.cn (J.L.); 18874583250@163.com (J.D.); 18373791197@163.com (Q.T.); yanchenyu20011016@163.com (C.Y.); asus43253@163.com (Y.L.); 2 Hunan Province Key Laboratory for Advanced Carbon Materials and Applied Technology, College of Materials Science and Engineering, Hunan University, Changsha 410082, China; yangjianxiao@hnu.edu.cn

**Keywords:** organophosphorus pesticides, guest-induced luminescent MOF, “turn-on” fluorescence sensor

## Abstract

Luminescent metal–organic frameworks (MOFs) are used for the detection of organophosphorus pesticides (OPs) due to their large surface area and pore volume as well as their special optical properties. However, most self-luminescent MOFs are not only complex to synthesize and unstable in water but also feature a “turn-off” sensing system, which has highly restricted their practical applications in OP detection. Herein, a “turn-on” fluorescence sensor based on the guest-induced luminescence MOF Ru(bpy)_3_^2+^@UiO-66 was constructed, which realized the sensitive detection of OPs through a dual-enzyme system for the first time. Compared with self-luminescent MOFs, Ru(bpy)_3_^2+^@UiO-66 was not only more easily synthesized but also had higher chemical and photostability in water. In this strategy, by means of the hydrolysis of AChE and ChOx, H_2_O_2_ will be produced, which can oxidize Fe^2+^ to Fe^3+^, thereby quenching the fluorescence of Ru(bpy)_3_^2+^@UiO-66. In the presence of OPs, the activity of AChE can be inhibited, resulting in the inability to generate H_2_O_2_ and Fe^3+^, which will turn on the fluorescence signal of Ru(bpy)_3_^2+^@UiO-66. As a result, the Ru(bpy)_3_^2+^@UiO-66 sensing system not only had high sensitivity for OPs detection but also possessed a satisfactory detection recovery rate for parathion-methyl in real samples, which provides a new approach for OP detection in food safety as well as environmental monitoring.

## 1. Introduction

Organophosphorus pesticides (OPs), a kind of organic pesticide containing phosphorus, are used to prevent and control plant diseases and insect pests due to their high efficiency [1]. However, the heavy use and improper treatment of OPs will lead to excessive residues in the environment, thus making water, soil and various crops polluted. In the meantime, they will be enriched in organisms through the food chain, causing pesticide poisoning [2,3]. It is universally recognized that the main reason for OP poisoning is that OPs can inhibit the activity of acetylcholinesterase (AChE) in the nervous tissues of organisms, which leads to the accumulation of the important neurotransmitter acetylcholine (ACh) in synapses and over-stimulation, resulting in central nervous injury [4,5]. Therefore, a serious threat is presented to human health by OP residues, which a variety of methods for OP detection have been developed to solve, such as high performance liquid chromatography (HPLC) [6,7], gas chromatography (GC) [8,9], enzyme-linked immunosorbent assay (ELISA) [10,11], electrochemical methods [12,13] and so forth. However, although HPLC and GC have good sensitivity, not only are expensive instruments but also complicated operations required. ELISA is susceptible to external environmental conditions and its required antibodies are difficult to prepare. In addition, electrochemical methods require complex treatment of the electrodes. Considering what is mentioned above, it is urgent to develop a sensitive and simple method to achieve efficient detection of OPs.

Currently, more and more attention is being attracted to fluorescence analysis, owing to its advantages of high sensitivity, quick response and simple operation. Meanwhile, many fluorescence assays based on enzyme inhibition have been developed to detect OPs [14,15,16,17,18,19,20,21,22]. Taking acetylcholinesterase (AChE) as an example, the main detection principle is that OPs can irreversibly inhibit the activity of AChE, leading to the failure of hydrolysis of the substrate ACh, so as to cause a change in the fluorescence intensity of probes for OP detection. For example, Li et al. [21] constructed a fluorescent sensor by using the FRET effect between NaY/GdF_4_:Yb, Er upconversion nanoparticles (UCNPs) and MnO_2_ nanosheets. Considering the effect of the inhibition of OPs on the activity of AChE, OP detection was realized by the change in the fluorescence intensity of UCNPs. However, the most common fluorescent probes used for detecting OPs have been mainly based on organic fluorescent dyes, UCNPs, quantum dots and so on. Owing to their complicated preparation process, instability and photobleaching, their practical applications are greatly limited. Therefore, research into a kind of highly efficient fluorescent probe for the determination of OPs is highly desirable.

Metal–organic frameworks (MOFs), a class of porous hybrid materials, self-assembled by metal nodes and organic ligands [23], have been used in many fields, including gas storage and separation [24,25], catalysis [26,27], electrochemistry [28,29], biomedicine [30,31] and fluorescence sensing [32,33] due to their advantages of high stability, easy surface modification, large surface area and pore volume. So far, some fluorescence sensors based on self-luminescent MOFs for OP detection have been developed. For instance, Xu et al. [34] synthesized a luminescent ZnPO-MOF. Through the photoinduced electron transfer (PET) effect between it and nitroaromatic compounds, the fluorescence of ZnPO-MOF would be suppressed by parathion-methyl, realizing the detection of parathion-methyl. However, the most self-luminescent MOFs were not only complex to synthesize and unstable in water but also featured a “turn-off” sensing system, which has highly restricted the practical application of MOFs in the detection of OPs. Meanwhile, many MOFs have a large specific surface area and pore volume as well as a stable crystal structure, and therefore, they can be used as excellent carriers of fluorescent guest molecules to construct novel fluorescent probes [35,36] and achieve sensitive detection of OPs in practice.

**Scheme 1:**
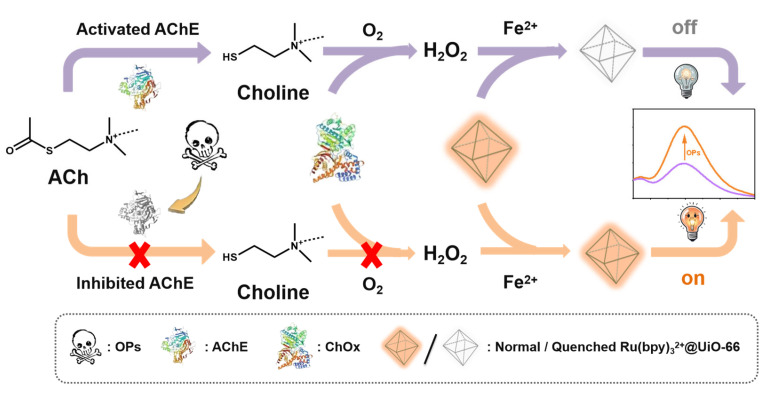
Schematic illustration of the principle of OP detection by using Ru(bpy)_3_^2+^@UiO-66.

In this work, a “turn-on” fluorescence sensor based on Ru(bpy)_3_^2+^@UiO-66 was designed for the detection of OPs, in which the fluorescence intensity of the Ru(bpy)_3_^2+^ molecule was greatly improved by the encapsulation of it into the UiO-66 framework. Furthermore, compared with self-luminescent MOFs, Ru(bpy)_3_^2+^@UiO-66 was not only synthesized more easily but also had higher chemical and photostability. In this sensing system, by means of the hydrolysis of the dual enzymes (AChE and ChOx), the generated choline can be transformed into H_2_O_2_, which can oxidize Fe^2+^ to Fe^3+^, quenching the fluorescence of Ru(bpy)_3_^2+^@UiO-66. When OPs appear, they can inhibit the activity of AChE, resulting in the inability to generate H_2_O_2_ and Fe^3+^, thus turning on the fluorescence of Ru(bpy)_3_^2+^@UiO-66 to achieve detection of OPs (Scheme 1), which provides a new strategy for the sensitive detection of OPs.

## 2. Results and Discussion

### 2.1. Characterization of UiO-66 and Ru(bpy)_3_^2+^@UiO-66

**Figure 1:**
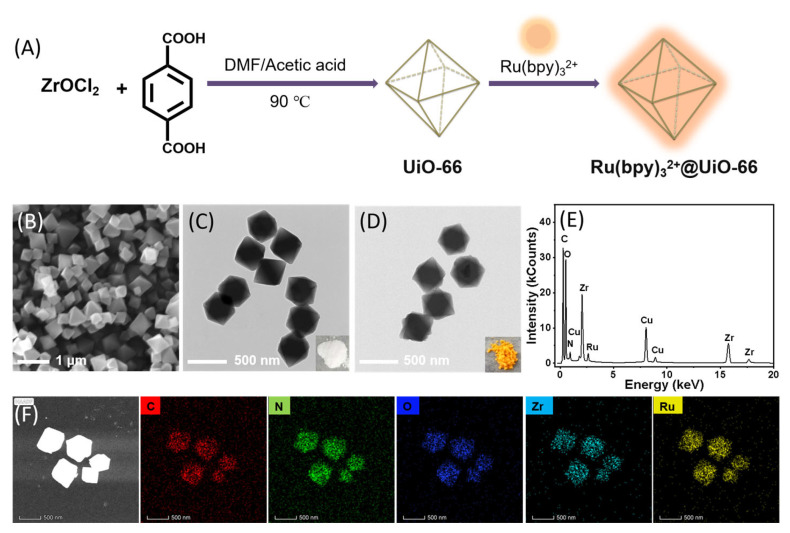
Synthesis of Ru(bpy)_3_^2+^@UiO-66 (**A**), SEM image of UiO-66 (**B**), TEM images of UiO-66 (**C**) and Ru(bpy)_3_^2+^@UiO-66 (**D**), EDX spectrum of Ru(bpy)_3_^2+^@UiO-66 (**E**) and HAADF-STEM image and elemental mappings of Ru(bpy)_3_^2+^@UiO-66 under high resolution, revealing elemental distribution (**F**). (Insets of (**C**,**D**) are photographs of corresponding powder taken under sunlight. Signals of Cu are from TEM grid in (**E**).)

As shown in the following SEM (Figure 1B) and TEM (Figure 1C) images, UiO-66, self-assembled from Zr^4+^ and terephthalic acid (Figure 1A), is an octahedral crystal with good dispersion and an average diameter of about 400 nm. By encapsulating the luminescent molecule Ru(bpy)_3_^2+^ into UiO-66, Ru(bpy)_3_^2+^@UiO-66 was successfully prepared (Figure 1A). Compared with UiO-66, the size and morphology of Ru(bpy)_3_^2+^@UiO-66 changed slightly, and its color changed from white to yellow (Figure 1D). In addition, the C, N, O, Zr and Ru were uniformly distributed within the Ru(bpy)_3_^2+^@UiO-66 framework, which was clearly demonstrated by the EDX spectrum (Figure 1E) and element mapping (Figure 1F).

**Figure 2:**
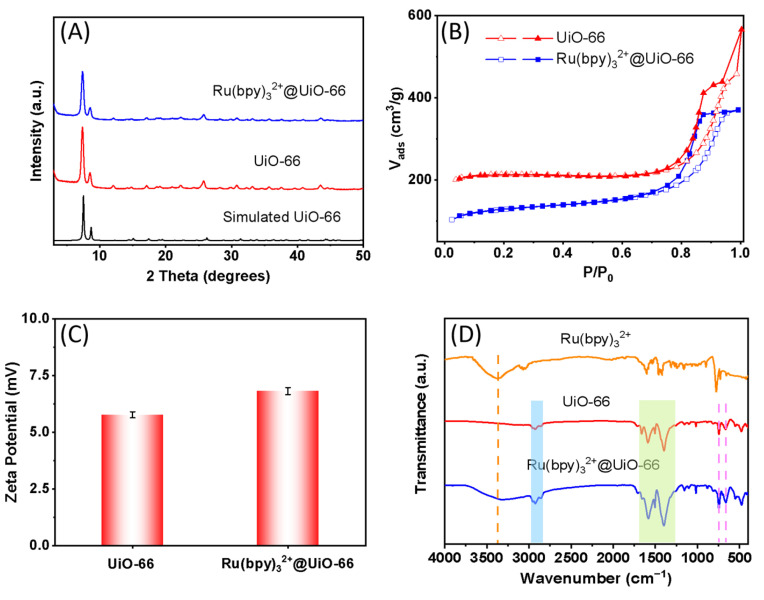
Powder X-ray diffraction (PXRD) profiles of the simulated UiO-66, synthesized UiO-66 and Ru(bpy)_3_^2+^@UiO-66 (**A**), N_2_ sorption isotherms (**B**) and zeta potentials (**C**) of UiO-66 and Ru(bpy)_3_^2+^@UiO-66, as well as the FT-IR spectra of Ru(bpy)_3_^2+^, UiO-66 and Ru(bpy)_3_^2+^@UiO-66 (**D**). (The BET surface areas and average pore sizes of UiO-66 and Ru(bpy)_3_^2+^@UiO-66 are 727.2 and 448.1 m^2^/g, as well as 1.6 and 1.4 nm, respectively. Filled and open symbols represent desorption and adsorption branches, respectively, in (**B**).)

Figure 2A shows that the PXRD patterns of the synthesized UiO-66 and Ru(bpy)_3_^2+^@UiO-66 are consistent with that of the simulated sample, implying the synthesized UiO-66 had bulky phase purity and the encapsulation of Ru(bpy)_3_^2+^ into it did not affect its crystallinity and structure. Additionally, compared with UiO-66, the decreased amount of N_2_ sorption (Figure 2B) and the increase in the ζ-potential (Figure 2C) of Ru(bpy)_3_^2+^@UiO-66 demonstrated that the Ru(bpy)_3_^2+^ luminophore was successfully introduced into the UiO-66 framework. FT-IR was also used to analyze the chemical functional groups of Ru(bpy)_3_^2+^@UiO-66. As illustrated in Figure 2D, the FT-IR spectrum of UiO-66 showed vibrational bands at 2970–2835 cm^−1^, ascribed to the C-H bonds, and strong bands at 1700–1250 cm^−1^, arisen from the carboxylate groups and phenyl ring deformations. Meanwhile, the bands at 745 and 665 cm^−1^ correspond to the Zr-O chemical bond [37]. Typically, the positions of these bands had hardly any change in the FT-IR spectrum of Ru(bpy)_3_^2+^@UiO-66, indicating that the structure of UiO-66 was not changed in Ru(bpy)_3_^2+^@UiO-66. In addition, there was a strong and wide band centered at 3370 cm^−1^ attributed to the O-H stretching mode of the physisorbed water in the spectrum of Ru(bpy)_3_^2+^, which also existed in the spectrum of Ru(bpy)_3_^2+^@UiO-66 [38]. All of these results further indicate that the Ru(bpy)_3_^2+^ molecule was successfully encapsulated by UiO-66. Then, ICP-AES was utilized to determine the loading capacity of the Ru complex in Ru(bpy)_3_^2+^@UiO-66. It was found that a high loading capacity of 10.35 wt% was obtained.

### 2.2. Optical Properties of Ru(bpy)_3_^2+^@UiO-66

**Figure 3:**
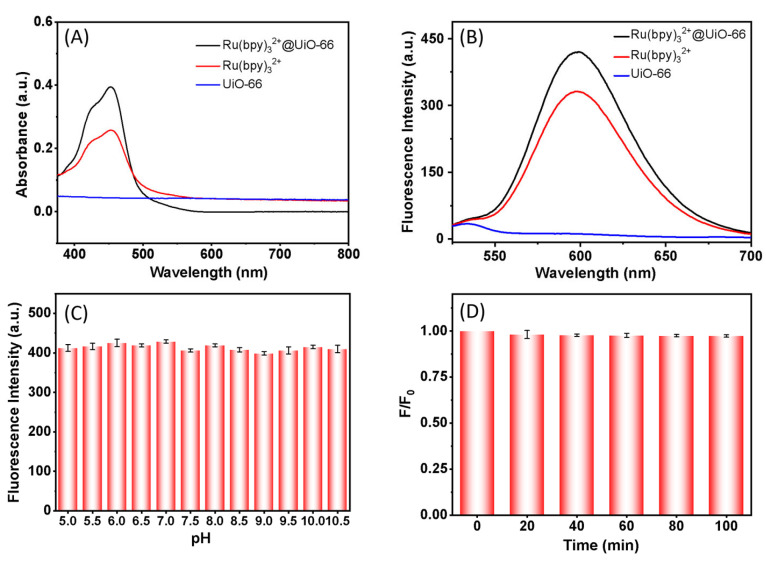
UV-vis absorption spectrum (**A**) and fluorescence spectra (λex = 450 nm) (**B**) of Ru(bpy)_3_^2+^, Ru(bpy)_3_^2+^@UiO-66 and UiO-66. Fluorescence intensity of Ru(bpy)_3_^2+^@UiO-66 at different pH values of PBS (**C**) and stability of photoluminescence of Ru(bpy)_3_^2+^@UiO-66 under UV lamp (**D**) (F_0_ and F represent the fluorescence intensity under UV lamp for 0 min and different times, respectively).

The basic optical properties of the probe were investigated. Compared with UiO-66, Ru(bpy)_3_^2+^ and Ru(bpy)_3_^2+^@UiO-66 exhibited obvious absorption peaks at 450 nm (Figure 3A) and strong fluorescence emission peaks at about 600 nm (Figure 3B), which indicated that the fluorescence signal of Ru(bpy)_3_^2+^@UiO-66 originated from the luminescent molecule Ru(bpy)_3_^2+^ rather than UiO-66, and further demonstrated that Ru(bpy)_3_^2+^ was encapsulated into the UiO-66 framework. In addition, since the porous structure of UiO-66 can not only separate the Ru(bpy)_3_^2+^ molecules to prevent aggregation-induced quenching but can also limit their motion or rotation to increase the rigidity of the Ru complex, the fluorescence intensity of Ru(bpy)_3_^2+^@UiO-66 was much stronger than that of the free Ru(bpy)_3_^2+^. It is well known that the chemical stability and photostability of the fluorescent probes are crucial for their practical applications. As displayed in Figure 3C, the fluorescence intensity of Ru(bpy)_3_^2+^@UiO-66 was almost constant in a wide pH range (5.0~10.5), implying that it has high chemical stability. Furthermore, it can be seen from Figure 3D that the fluorescence intensity of Ru(bpy)_3_^2+^@UiO-66 hardly decreased with the increasing time of UV lamp irradiation. The above results indicate that Ru(bpy)_3_^2+^@UiO-66 has good chemical and photostability, which provides a reliable guarantee for the construction of sensitive OP sensors.

**Figure 4:**
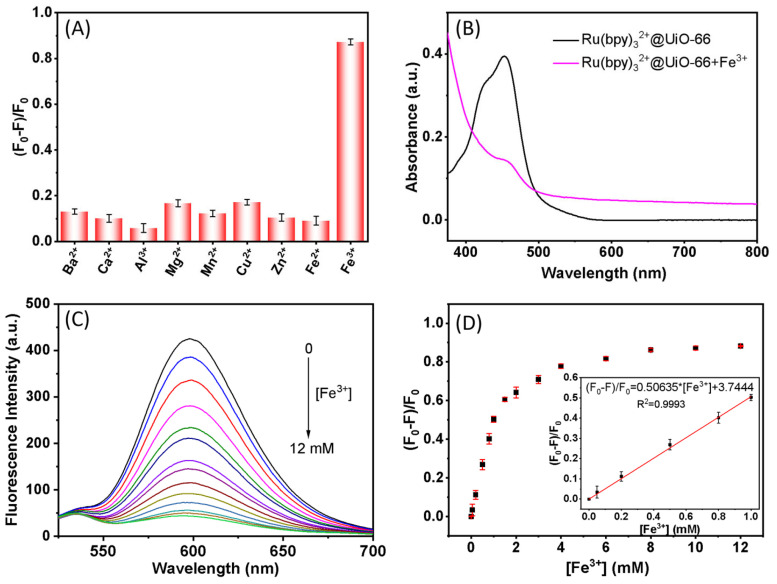
Fluorescence quenching rate of Ru(bpy)_3_^2+^@UiO-66 in the presence of different metal ions. (The concentration of the metal ions was 10 mM. F_0_ and F represent the fluorescence intensity of Ru(bpy)_3_^2+^@UiO-66 before and after adding metal ions, respectively.) (**A**) The UV-vis absorption spectrum of Ru(bpy)_3_^2+^@UiO-66 and Ru(bpy)_3_^2+^@UiO-66+Fe^3+^ (**B**), fluorescence spectra (λex = 450 nm) of Ru(bpy)_3_^2+^@UiO-66 (10 μg/mL) in the presence of various concentrations of Fe^3+^ (0, 0.05, 0.2, 0.5, 0.8, 1, 1.5, 2, 3, 4, 6, 8, 10 and 12 mM) (**C**) and the corresponding linear plot of (F_0_ − F)/F_0_ versus the concentration of Fe^3+^ (**D**).

Subsequently, the effects of different metal ions (Ba^2+^, Ca^2+^, Al^3+^, Mg^2+^, Mn^2+^, Cu^2+^, Zn^2+^, Fe^2+^ and Fe^3+^) on the fluorescence intensity of Ru(bpy)_3_^2+^@UiO-66 were explored. Figure 4A implies that the fluorescence quenching rate of Ru(bpy)_3_^2+^@UiO-66 by Fe^3+^ was as high as 87.28% after incubation for 10 min, while those of other metal ions were very low, suggesting Fe^3+^ was able to quench the fluorescence signal of Ru(bpy)_3_^2+^@UiO-66, which may be because Fe^3+^ has strong oxidizability and simple coordination in the environment of an aqueous solution, which enables it to efficiently quench the fluorescence of Ru(bpy)_3_^2+^ through the photoinduced electron transfer (PET) effect. Specifically, compared with other common transition metal ions, Fe^3+^ is one of the strongest oxidants because the Fe^3+^/Fe^2+^ pair has a very positive standard reduction potential (+0.77 V). In addition, Fe^3+^, existing in the form of [Fe(H_2_O)_6_]^3+^ in aqueous solution, is a relatively “hard” Lewis acid, which means that its dynamic electron transfer does not require coordination layer recombination, and its rate is fast. Therefore, compared to other transition metal ions, Fe^3+^ can efficiently quench the fluorescence signal of Ru(bpy)_3_^2+^@UiO-66 in a short time. Furthermore, in order to further investigate the recognition behavior of Ru(bpy)_3_^2+^@UiO-66 on Fe^3+^, the UV-vis absorption spectra of this system were measured. Figure 4B shows that the intensity of the absorption peak of Ru(bpy)_3_^2+^@UiO-66 obviously decreased when Fe^3+^ was introduced, which is mainly due to the ground state interaction between Fe^3+^ and Ru(bpy)_3_^2+^, forming a ground state complex that has almost no absorption at 450 nm. Subsequently, the quenching effect of different concentrations of Fe^3+^ on the fluorescence intensity of Ru(bpy)_3_^2+^@UiO-66 was studied. As shown in Figure 4C, with the increase of Fe^3+^ concentration, the fluorescence intensity of Ru(bpy)_3_^2+^@UiO-66 gradually decreased, and there is a good linear relationship between the fluorescence quenching rate ((F_0_ − F)/F_0_) and the concentration of Fe^3+^ in the range of 0~1.0 mM [(F_0_ − F)/F_0_ = 0.50635 [Fe^3+^] + 3.7444, R^2^ = 0.9993], with a detection limit of 0.038 mM (Figure 4D).

### 2.3. Feasibility of Ru(bpy)_3_^2+^@UiO-66 Sensor for OP Sensing

A “turn-on” fluorescence sensor based on the guest-induced luminescent Ru(bpy)_3_^2+^@UiO-66 was developed to detect OPs, the basic mechanism of which includes three parts, as follows. (1) AChE catalyzes the hydrolysis of substrate ACh to produce choline (Equation (1)), and then choline and O_2_ are hydrolyzed by ChOx to betaine and H_2_O_2_ (Equation (2)) [39,40]. (2) Fe^2+^ is oxidized to Fe^3+^ by H_2_O_2_ (Equation (3)) [41]. (3) The generated Fe^3+^ can quench the fluorescence of Ru(bpy)_3_^2+^@UiO-66, while, in the presence of OPs, the activity of AChE is inhibited by it. Therefore, the choline, H_2_O_2_ and final Fe^3+^ will not be able to generate, thus triggering the fluorescence of Ru(bpy)_3_^2+^@UiO-66 to achieve detection of OPs (Scheme 1).

(1) $$ACh+H_{2}O\overset{AChE}{\to }\;\mathrm{choline}\;+\;\mathrm{acetic}\;\mathrm{acid}\;$$

(2) $$\;\mathrm{choline}\;+O_{2}\overset{ChOx}{\to }\;\mathrm{betaine}\;+H_{2}O_{2}$$

(3) $${Fe}^{2+}+H_{2}O_{2}\overset{H^{+}}{\to }{Fe}^{3+}+H_{2}O$$

**Figure 5:**
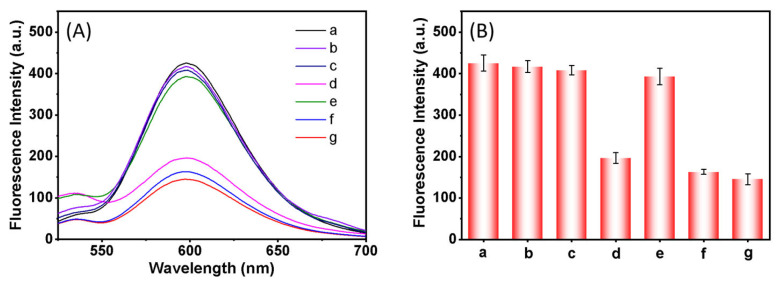
The effects of different substances on the fluorescence intensity of Ru(bpy)_3_^2+^@UiO-66. The fluorescence emission spectra (λex = 450 nm) (**A**) and the corresponding fluorescence intensity (**B**) of Ru(bpy)_3_^2+^@UiO-66 in different substances. [Ru(bpy)_3_^2+^@UiO-66 (a), Ru(bpy)_3_^2+^@UiO-66 + AChE + ChOx (b), Ru(bpy)_3_^2+^@UiO-66 + AChE + ChOx + ACh (c), Ru(bpy)_3_^2+^@UiO-66 + AChE + ChOx + ACh + Fe^2+^ (d), Ru(bpy)_3_^2+^@UiO-66 + AChE + ChOx + ACh + Fe^2+^ + parathion-methyl (e), Ru(bpy)_3_^2+^@UiO-66 + Fe^2+^ + H_2_O_2_ (f) and Ru(bpy)_3_^2+^@UiO-66 + Fe^3+^ (g)].

Figure 5 shows the effects of different substances on the fluorescence intensity of Ru(bpy)_3_^2+^@UiO-66, which indicate that the fluorescence was significantly quenched only when Ru(bpy)_3_^2+^@UiO-66 coexisted with AChE, ChOx, ACh and Fe^2+^ (d), H_2_O_2_ and Fe^2+^ (f) and Fe^3+^ (g). In comparison, other conditions had little effect on it. The results demonstrated the above mechanism of this strategy, in which only in the presence of double enzymes (AChE and ChOx) would H_2_O_2_ be produced and further oxidize Fe^2+^ into Fe^3+^, thus quenching the fluorescence of Ru(bpy)_3_^2+^@UiO-66 (Equations (1)–(3)). However, in the presence of OPs, the activity of AChE was inhibited, and H_2_O_2_ could not be generated to convert Fe^2+^ to Fe^3+^, resulting in the turning-on of this fluorescence system.

### 2.4. Detection of OPs

**Figure 6:**
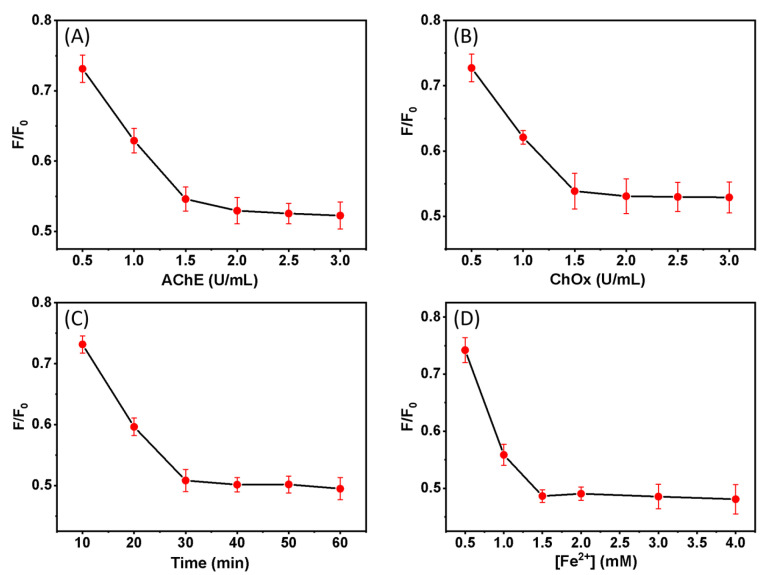
The relative fluorescence intensity (F/F_0_) under different concentrations of AChE (0.5, 1.0, 1.5, 2.0, 2.5 and 3.0 U/mL) (**A**), ChOx (0.5, 1.0, 1.5, 2.0, 2.5 and 3.0 U/mL) (**B**) and Fe^2+^ (0.5, 1.0, 1.5, 2.0, 3.0 and 4.0 mM) (**D**), and under different incubate times (10, 20, 30, 40, 50 and 60 min) of the AChE + ACh + ChOx system (**C**).

In order to realize the sensitive detection of OPs, the concentrations of ATCh, ChOx and Fe^2+^, as well as the incubation time, were systematically optimized. As displayed in Figure 6A,B, the fluorescence intensity of Ru(bpy)_3_^2+^@UiO-66 basically remained constant when the concentrations of AChE and ChOx reached 2.0 U/mL and 1.5 U/mL, respectively. Thus, 2.0 U/mL and 1.5 U/mL were chosen as the optimal concentrations of AChE and ChOx, respectively. Subsequently, the incubation times of AChE, ACh and ChOx were studied. Figure 6C shows that the fluorescence intensity of this system reached a balance after incubation for 30 min. Furthermore, the concentration of Fe^2+^ in the system was investigated. It can be seen from Figure 6D that with the increasing concentration of Fe^2+^, the relative fluorescence intensity attained a plateau at 1.5 mM; therefore, the optimum concentration of Fe^2+^ was 1.5 mM in this sensing system.

**Figure 7:**
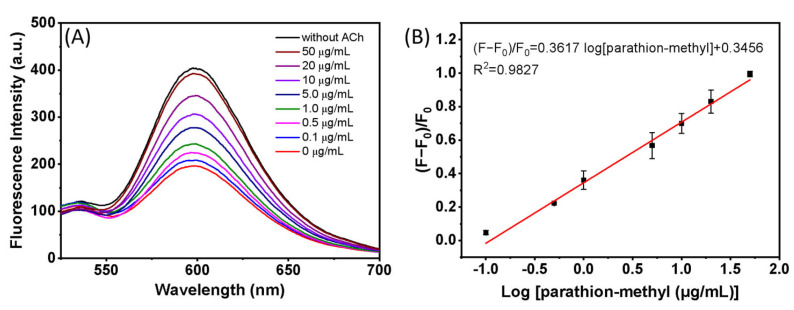
The fluorescence spectra (λex = 450 nm) of AChE + ACh + ChOx + Ru(bpy)_3_^2+^@UiO-66 + Fe^2+^ in the presence of various concentrations of parathion-methyl (0, 0.1, 0.5, 1.0, 5.0, 10, 20, 50 μg/mL) (**A**) and the linear plot of (F−F_0_)/F_0_ versus the logarithm of parathion-methyl concentration (**B**) (F and F_0_ are the fluorescence intensity of the Ru(bpy)_3_^2+^@UiO-66 system in the presence and absence of parathion-methyl.) Ru(bpy)_3_^2+^@UiO-66: 10 μg/mL; [ACh]: 40 mM; [AChE]: 2 U/mL; [ChOx]: 1.5 U/mL; [Fe^2+^]: 1.5 mM; PBS (10 mM, pH 7.5).

Under the optimum conditions, the detection of parathion-methyl, which is one of the widely used OPs, by the Ru(bpy)_3_^2+^@UiO-66 sensing system was investigated. From Figure 7A, we can see that with the increase in the concentration of parathion-methyl, the fluorescence intensity of Ru(bpy)_3_^2+^@UiO-66 gradually increased, which indicates that the inhibition of the activity of AChE by parathion-methyl suppresses the generation of Fe^3+^, thus turning on the fluorescence of Ru(bpy)_3_^2+^@UiO-66. In addition, the inhibition rate ((F−F_0_)/F_0_, F_0_ and F, corresponding to the fluorescence intensity of Ru(bpy)_3_^2+^@UiO-66 in the absence and presence of parathion-methyl, respectively) had a linear relationship with the logarithm of parathion-methyl concentration from 0.1 to around 50.0 μg/mL, and the corresponding linear regression equation was (F−F_0_)/F_0_ = 0.3617 log [parathion-methyl] + 0.3456, with the limit of detection (LOD) at 10.6 ng/mL, which was lower than those of most other reported fluorescence sensors (Table 1).

### 2.5. Anti-Interference Ability, Selectivity and Recyclability

**Figure 8:**
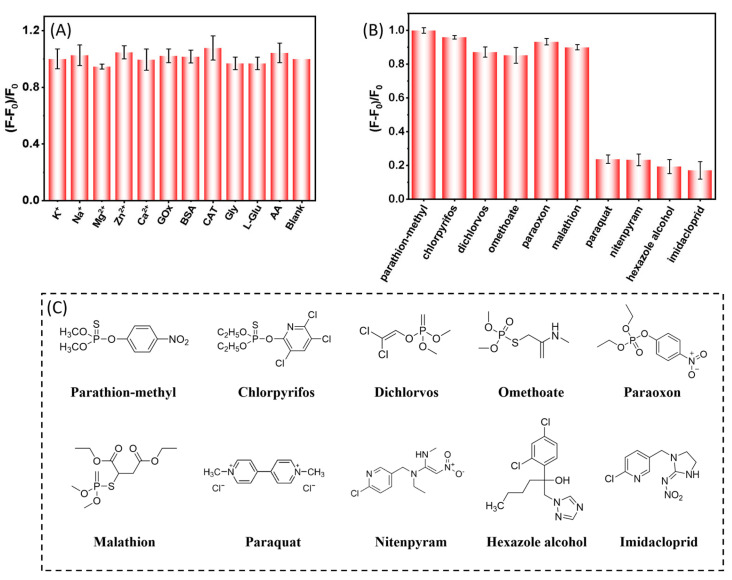
The inhibition rate ((F−F_0_)/F_0_) of the parathion-methyl + AChE + ACh + ChOx + Ru(bpy)_3_^2+^@UiO-66 + Fe^2+^ system with different interfering substances (**A**) and 50 μg/mL different pesticides on the AChE+ACh+ChOx+Ru(bpy)_3_^2+^@UiO-66 + Fe^2+^ system (**B**), as well as the chemical structures of the various pesticides (**C**).

In order to explore the anti-interference ability of the proposed sensor, the effects of common metal ions and bioelectrolytes in food samples on this sensor were investigated. It can be seen from Figure 8A that Ca^2+^, K^+^, Mg^2+^, Na^+^, Zn^2+^, glycine (Gly), ascorbic acid (AA), bovine serum albumin (BSA), catalase (CAT), glucose oxidase (GOx) and L-glutamic acid (L-Glu) had little effect on the detection of parathion-methyl. In addition, to prove the selectivity of the Ru(bpy)_3_^2+^@UiO-66 fluorescence sensing system for the detection of parathion-methyl, the effects of five common OPs (chlorpyrifos, dichlorvos, omethoate, paraoxon and malathion) and four other kinds of pesticides (paraquat, nitenpyram, hexazolalcohol and imidacloprid) on the system were studied. As can be seen from Figure 8B, all of the OPs displayed an inhibition effect on the system, with parathion-methyl displaying the highest inhibitory rate, while the other kinds of pesticides exerted a negligible effect to the system, which is principally because the Ops, owning similar chemical structures (Figure 8C), can trigger the irreversible inhibition of AChE. Therefore, the proposed Ru(bpy)_3_^2+^@UiO-66 fluorescence sensing system exhibits excellent selectivity for OPs, and can thus be used as a universal sensing platform for identification and detection of OPs. Furthermore, the eight cycles of detection of 10 μg/mL parathion-methyl by the Ru(bpy)_3_^2+^@UiO-66 sensing system showed no significant fluorescent variation, and the relative standard deviation (RSD) was 4.3%, indicating the excellent recyclability of the Ru(bpy)_3_^2+^@UiO-66 system.

### 2.6. Detection of OPs in Actual Samples

Parathion-methyl in environmental and agricultural samples (tap water, lake water and cabbage) was detected via the standard addition method. As shown in Table 2, the recovery was between 92.80% and 106.8%, and the RSD was less than 5.6%. The results indicated that the Ru(bpy)_3_^2+^@UiO-66 sensing system possessed an excellent ability to detect OPs in actual samples.

## 3. Materials and Methods

### 3.1. Reagents and Instruments

Acetylcholinesterase (AChE, 500 units, derived from electric eel) and choline oxidase (ChOx, 100 units, derived from Alcaligenes) were purchased from Sigma-Aldrich Co., Ltd. (St. Louis, MO, USA). Ammonium ferrous sulfate [(NH_4_)_2_Fe(SO_4_)_2_·6H_2_O], terephthalic acid (PTA), zirconium oxychloride (ZrOCl_2_·8H_2_O), acetylcholine chloride (ACh), ruthenium chloride hexahydrate (Ru(bpy)_3_Cl_2_·6H_2_O, >98%), parathion-methyl, dichlorvos, chlorpyrifos, imidacloprid and hexazole alcohol were obtained from Aladdin Biochemical Technology Co., Ltd. (Shanghai, China). Malathion, omethoate, paraquat, nitenpyram and paraoxon were purchased from Walbo Technology Co., Ltd. (Beijing, China). N, N-dimethylformamide (DMF) and glacial acetic acid were provided by Sinopharm Chemical Reagent Co., Ltd. (Shanghai, China). All reagents were used without any further purification.

Scanning electron microscopy (SEM, SU8010, Hitachi, Tokyo, Japan), transmission electron microscopy (TEM, TalosF200X) and high-angle annular dark-field scanning TEM (HAADF-STEM) images with an energy-dispersive X-ray detector (EDX) were used for the morphological characterization and detailed composition analysis of the prepared samples. Powder X-ray diffraction (PXRD) measurements were performed on a D8-ADVANCE powder diffractometer (Brooke, Karlsruhe, Germany). The nitrogen adsorption–desorption isotherms were obtained using a Micromeritics ASAP 2460 Sorptometer (Mike Instruments, Cincinnati, OH, USA). A Zeta potentiometer (Zetasizer Nano ZS90, Malvern Instruments, Malvern, UK) was used to measure the surface potential of the samples. Nicolet iS10 Fourier transform infrared spectroscopy (FT-IR, Thermo Fisher Scientific, Waltham, MA, USA) was employed to characterize the chemical functional groups of the samples. Inductively coupled plasma atomic emission spectroscopy (ICP-AES) was carried out on a PerkinElmer ICP-AES system (PE 2100DV, Waltham, MA, USA). The UV-Vis spectra were collected by using a UV-2700 spectrophotometer (Shimadzu, Kyoto, Japan). The fluorescence emission spectra were recorded via an F-7000 fluorescence spectrophotometer (Hitachi, Japan). Ultrapure water (18.2 MΩ·cm^−1^) for the sample preparation and detection was prepared with the Milli-Q system (Millipore, Bedford, MA, USA).

### 3.2. Synthesis of UiO-66

UiO-66 was synthesized according to the reported literature [51]. First, ZrOCl_2_ (21 mg, 0.066 mM) and terephthalic acid (50 mg, 0.24 mM) were dissolved into 3 mL and 1 mL DMF, respectively. Then, the above two solutions were added into 400 μL glacial acetic acid and mixed and heated at 90 °C for 18 h. After centrifugation, the mixture was washed with DMF and ultrapure water, respectively. Finally, the obtained solid was dried under vacuum for 12 h at 60 °C for future use.

### 3.3. Preparation of Ru(bpy)_3_^2+^@UiO-66

Ru(bpy)_3_^2+^@UiO-66 was prepared as follows. First, 20 mg of the synthesized UiO-66 was dispersed into 10 mL DMF. Then, the above suspension was mixed with a 10 mL DMF solution containing Ru(bpy)_3_Cl_2_·6H_2_O (0.5, 1 or 2 mg/mL). Subsequently, the mixture was stirred for 12 h at 90 °C and washed with DMF and ethanol, respectively, after centrifugation. Finally, the yellow solid was dried under vacuum at 60 °C for 12 h to obtain Ru(bpy)_3_^2+^@UiO-66.

ICP-AES was utilized to determine the loading capacity of the Ru complex in Ru(bpy)_3_^2+^@UiO-66. Since the results indicated that 1 mg/mL of Ru(bpy)_3_Cl_2_·6H_2_O achieved the highest loading capacity (10.35 wt%), all subsequent experiments were conducted using Ru(bpy)_3_^2+^@UiO-66 prepared at this concentration.

### 3.4. Fluorometric Detection of OPs

The Ru(bpy)_3_^2+^-UiO-66 was dispersed in deionized water as a stock solution (1 mg/mL), and acetonitrile was used as the solvent for the standard stock solution of the parathion-methyl. The solutions of various biological species (such as AChE, ChOx and so on) were dissolved or diluted with PBS buffer solution (10 mM, pH 7.5). Firstly, different concentrations of parathion-methyl (0, 0.1, 0.5, 1, 5, 10, 20 and 50 μg/mL) were incubated with 200 μL AChE (2 U/mL) for 15 min. Next, 500 μL ACh (40 mM) and 500 μL ChOx (1.5 U/mL) were added into the above solution and incubated at 37 °C for another 30 min. Then, 300 μL the above solution, 10 μL Ru(bpy)_3_^2+^-UiO-66 (1 mg/mL), 200 μL HCl (3%), 365 μL PBS (10 mM, pH 7.5) and 125 μL Fe^2+^ (1.5 mM) were mixed thoroughly for 10 min before the fluorescence spectra were collected at an excitation wavelength of 450 nm.

### 3.5. OP Detection in Real Samples

This sensor was mainly used for the detection of parathion-methyl in environmental and food samples, including lake water, tap water and cabbage samples. The insoluble materials in the lake water sample (10 mL) were removed by the 0.22 μm filter membrane and the chlorine was evaporated from the tap water sample (10 mL) by boiling. Then, the treated water samples were mixed with 10 mL PBS (10 mM, pH 7.4) containing 20% methanol, respectively. As for the cabbage, it (10 g) was ground and mixed with 50 mL acetonitrile under sonication for 10 min. Then, the mixture was centrifuged to obtain the supernatant. The extraction process was repeated three times, and the extraction solutions were combined and evaporated to dry, then dissolved by PBS (10 mM, pH 7.4) containing 20% methanol. Subsequently, the standard solution of 5, 10 and 15 μg/mL parathion-methyl was spiked into the three samples. The recovery (%) and relative standard deviation (RSD) (*n* = 3, %) were calculated to evaluate the practical value of the sensor.

## 4. Conclusions

To sum up, a “turn on” fluorescence sensor based on the guest-induced luminescence Ru(bpy)_3_^2+^@UiO-66 was constructed and it realized the sensitive detection of OPs for the first time. In this strategy, the fluorescence of Ru(bpy)_3_^2+^@UiO-66 was quenched by Fe^3+^ which was generated by the dual enzymes and Fe^2+^ system. In the presence of OPs, they can inhibit the activity of AChE, thereby lighting the fluorescence signal of Ru(bpy)_3_^2+^@UiO-66 and achieving OPs detection. The experimental results implied a good linear relationship with the logarithm of the parathion-methyl concentration from 0.1 μg/mL to 50.0 μg/mL, and the LOD at 10.6 ng/mL. Furthermore, the recovery was between 92.80% and 106.8% for the detection of actual samples. Therefore, this method opened up a new avenue for the detection of OPs.

## Figures and Tables

**Table 1:** Comparison of various fluorescence probes for detecting parathion-methyl.

Probe	Linear Range (μg/mL)	Detection Limit (ng/mL)	Reference
THIP-OCP	0–450.0	790.0	[14]
Cu^2+^/TAPB-DMTA-COF	0.1474–1.437	8.080	[42]
β-CD-MoS_2_ QDs	0.05000–25.00	32.00	[43]
CdTe QDs	0.02500–3.000	18.00	[44]
bCDs	0.07896–17.11	36.85	[45]
CQDs-NFP	0.005000–14.00	--	[46]
g-C_3_N_4_/AgNPs	100.0–1000	3.240 × 10^4^	[47]
N-doped carbon dots	626.4–1.942 × 10^4^	8.900 × 10^4^	[48]
OPAA-CQDs	0–26.30	684.4	[49]
N-CDs-DAP	--	13.00	[50]
Ru(bpy)_3_^2+^@UiO-66	0.1000–50.00	10.60	This work

**Table 2:** Determination of parathion-methyl from water and agricultural samples by this strategy.

Sample	Spiked (μg/mL)	Found (μg/mL)	Recovery (%)	RSD (*n* = 3, %)
Lake water	0	Not found	—	—
	5.000	5.340	106.8	3.1
	10.00	10.34	103.4	4.3
	15.00	15.07	100.5	4.7
Tap water	0	Not found	—	—
	5.000	4.640	92.80	2.9
	10.00	10.54	105.4	2.4
	15.00	14.78	98.53	4.3
Cabbage	0	Not found	—	—
	5.000	4.680	93.60	5.6
	10.00	10.06	100.6	1.0
	15.00	15.04	100.3	4.2

## Data Availability

The data are contained within the article.
